# Correlation Between the Pitch and Loudness of Tinnitus, Hearing Levels, and Tinnitus Handicap Inventory Scores in Patients with Chronic Subjective Tinnitus

**DOI:** 10.3390/jcm13237261

**Published:** 2024-11-29

**Authors:** Panayiota Mavrogeni, András Molnár, Viktória Molnár, László Tamás, Stefani Maihoub

**Affiliations:** 1 Tóth Ilona Health Service Clinical Medical Institute, H-1212 Budapest, Hungary; panayiota.mavrogeni@icloud.com; 2 Department of Otorhinolaryngology and Head and Neck Surgery, Semmelweis University, Szigony u. 36, H-1083 Budapest, Hungary; 3 Department of Voice, Speech and Swallowing Therapy, Semmelweis University, Vas u. 17, H-1088 Budapest, Hungary

**Keywords:** tinnitus pitch, tinnitus loudness, hearing level, hearing loss frequency, tinnitus handicap inventory

## Abstract

**Objectives:** The aim of this study was to investigate the relationships between tinnitus pitch and loudness, the frequency of hearing loss, hearing level, and the Tinnitus Handicap Inventory (THI) scores reported by patients. **Methods:** In total, 139 patients (50 men and 89 women; mean age ± SD, 60.19 ± 11.47 years) who suffered from chronic primary tinnitus associated with sensorineural hearing loss were included in the study. Participants underwent pure-tone audiometry and tinnitus pitch matching assessments. Additionally, all participants completed the Hungarian version of the THI questionnaire. Correlations were assessed using simple linear, Spearman’s, and Pearson’s correlation tests, and a linear regression model was applied. The Mann–Whitney *U* test was also used. **Results:** This study identified a significant correlation between the pitch of tinnitus and the frequency of hearing loss (*p* = 0.000 *; rho = 0.549). There was also a significant correlation between tinnitus noise and hearing levels (*p* = 0.000 *; rho = 0.375). Age was shown to significantly affect tinnitus loudness (*p* = 0.016 *) and hearing levels (*p* = 0.000 *) as determined by a linear regression model. Tinnitus duration only significantly influenced tinnitus loudness (*p* = 0.022). There was no significant effect of sex on tinnitus or audiometry parameters. Total THI scores were influenced solely by tinnitus loudness (*p* = 0.021 *). Furthermore, sex did have an effect on total THI scores, with women reporting higher scores (*p* = 0.000 *). **Conclusions:** This study concluded that there is a significant correlation between the pitch and loudness of tinnitus and hearing levels, suggesting a connection in their underlying mechanisms. The intensity of tinnitus and hearing level are primarily affected by ageing processes. Furthermore, the severity of self-perceived tinnitus is mainly related to the loudness of tinnitus.

## 1. Introduction

Tinnitus is the perception of sound without an external source, and it is typically only heard by the person experiencing it, meaning that it is subjective [1]. An estimated 10–15% of adults suffer from tinnitus [2]. Tinnitus can be categorised as either primary or secondary, with primary cases including tinnitus linked to sensorineural hearing loss [3]. The potential causes of tinnitus combined with sensorineural hearing loss include exposure to noise, ageing (i.e., presbycusis), and ototoxicity [4,5]. The relationship between tinnitus parameters and audiometric profiles has been extensively researched. Two neurophysiological models have been proposed to explain their potential association. The tonotopic model suggests that tinnitus may result from a reorganisation in the central auditory system following a hearing loss. This reorganisation may cause the cortical areas to become attuned to the frequency spectrum of hearing loss, which is referred to as the ‘edge’ frequency. As a result, because the edge frequency is overrepresented, the perception of tinnitus aligns with this edge frequency [6]. However, according to the neural synchrony model, the pitch of tinnitus may arise from spontaneous neural hyperactivity in the area affected by hearing loss [7]. The homeostatic plasticity model indicates that increased neuronal activity serves as a compensatory mechanism to stabilise neural activities following hearing loss [8]. The existing literature presents conflicting data about the relationship between tinnitus pitch and audiometric results. Numerous studies have shown a correlation between hearing loss and tinnitus pitch, suggesting that tinnitus can be identified by localising hearing loss [9]. Furthermore, it appears that around 80–93% of tinnitus patients also experience hearing loss [10]. Thus, evaluating the hearing function of all tinnitus sufferers is imperative [5,11].

Previous studies have shown that tinnitus can significantly affect a person’s daily functioning and overall quality of life. For example, it has been reported that tinnitus may reduce the quality of life in adults by as much as 5% [12]. Moreover, previous studies have found that tinnitus can have a significant impact on patients’ daily lives and quality of life, as evaluated by tools like the Tinnitus Handicap Inventory (THI) questionnaires [13]. The use of self-administered questionnaires (e.g., THI or TFI—Tinnitus Functional Index) is crucial for evaluating the severity and impact of tinnitus on daily functioning, as the way individuals perceive tinnitus can differ. For instance, a previous study indicated that tinnitus may worsen when it occurs alongside hearing loss [14]. However, another study has not detected a significant correlation between THI and audiometric characteristics [15].

Consequently, using the THI questionnaires, the present investigation aims to describe and examine the correlation between audiometric and tinnitus characteristics and patients’ subjective reports.

## 2. Materials and Methods

### 2.1. Study Sample

This study included 139 participants, comprising 50 men and 89 women, with an average age of 60.19 ± 11.47 years. All participants experienced chronic subjective tinnitus and sensorineural hearing loss in the same ear. Other symptoms, including aural fullness, vertigo, dizziness, and headache, were evaluated and excluded through detailed questioning. The study specifically focused on individuals experiencing persistent subjective tinnitus, lasting for more than six months (i.e., chronic tinnitus). Additionally, individuals with secondary tinnitus—such as those suffering from inflammations of the external or middle ear, Ménière’s disease, temporomandibular joint disorders, vascular abnormalities, Eustachian tube dysfunction, or palatal and middle ear myoclonus—were excluded from this study. Furthermore, individuals diagnosed with psychiatric, neurologic, or cardiovascular disorders were excluded from the study. Secondary tinnitus cases were eliminated through comprehensive examinations that included physical assessments, medical imaging, laboratory tests, blood pressure monitoring, ECG, and, when necessary, cardiological evaluations. Additional examinations may be required based on the patients’ symptoms and the characteristics of their tinnitus. Patients with incomplete medical records were also excluded. A neurological examination ruled out any neurological issues, and each individual underwent a brain MRI. Psychiatric symptoms were typically evaluated using questionnaires. If signs of possible psychiatric problems were present, a psychiatric evaluation was conducted. The distribution of symptom locations was as follows: right-sided (*n* = 21, 15%), left-sided (*n* = 49, 35%), and bilateral (*n* = 69, 50%). The mean ± SD duration from the first onset of tinnitus was defined as 49.1 ± 35.61 months. All participants received a comprehensive examination of their ears, nose, throat, larynx, and neck. In addition, nasal endoscopy, micro-otoscopy, tympanometry, and acoustic reflex testing were performed. An audiological examination was conducted, as outlined below. Acoustic neuroma (vestibular schwannoma) and other central abnormalities were excluded through a brain MRI. The Hungarian version of the THI was used to assess the impact of tinnitus on the participants. All participants provided written informed consent. All patients were examined by the same specialist in otorhinolaryngology and audiology (P.M.) at the Tóth Ilona Health Service Clinical Medical Institute in Budapest, Hungary.

### 2.2. Audiological Examinations

Prior to the audiological examination, all participants underwent otoscopy and tympanometry (Flute inventis, ANSI S3.39–1987 type 2, Padova, Italy) to assess the status of their external and middle ears. If any pathologies were detected, audiological examinations were either not performed or were interrupted. Pure-tone audiometry was conducted using a GSI 61 Clinical Audiometer (Granson-Stadler, Inc., Milford, CT, USA) in a soundproof booth. The patient was seated in a double-walled, sound-treated room. Pure-tone air conduction (125–8000 Hz) and bone conduction (250–4000 Hz) were measured in all participants using headphones and a mastoid vibrator, respectively. In necessary cases, masked bone conduction testing was utilised. The right ear was tested first consistently. The modified Hughson–Westlake procedure [16] was used to perform the audiometry. This involved identifying the lowest sound intensities perceivable in both ears, utilising octave and inter-octave frequencies in 5 dB increments [16]. Standard manual audiometry was employed to measure the testing thresholds. Pure-tone averages were calculated, and sensorineural hearing loss was defined according to the 1995 recommendations of the Committee on Hearing and Equilibrium of the American Academy of Otolaryngology–Head and Neck Surgery [17].

All participants underwent testing tlo determine the pitch and loudness of their tinnitus. The examinations focused on the right ear, left ear, or both, depending on where the symptoms were located. Tinnitus pitch matching was performed using frequencies that ranged from 125 Hz to 8000 Hz, employing a ‘bracketing’ method [18] that began at 1000 Hz. Participants were asked to indicate whether the sound played was higher or lower than their perceived tinnitus level. When the sound stimulus was higher than the participant’s tinnitus, it was adjusted by half an octave. Matching was halted when the frequency equalled the perceived tinnitus frequency, as reported by the patients. The final selected pitch for the tinnitus was confirmed using the octave confusion test, which compared the frequency of the chosen tinnitus with frequencies one octave higher and one octave lower [19]. This procedure involved using frequencies that were one octave below and one octave above the selected tinnitus pitch, with each of the three frequencies presented at the same intensity level. The participant was then asked to select one of the three frequency options, and their choice was recorded as the final frequency determined in the pitch matching process. Subsequently, the loudness of the tinnitus was measured at a specific frequency using tinnitus pitch matching. The sound stimulus was adjusted in small increments (e.g., 1 dB), to match the tinnitus loudness as accurately as possible. Both matching procedures were conducted three times using a forced choice method. The pitch and loudness of the tinnitus were then manually indicated on the audiogram thresholds.

### 2.3. Tinnitus Handicap Inventory

The previously validated Hungarian version of the THI questionnaire was utilised to evaluate tinnitus severity based on patients’ subjective reports [20,21]. The THI comprises 25 questions grouped into emotional (*E*), functional (*F*), and catastrophic (*C*) categories. The ‘*E*’ category comprises nine questions related to issues such as anxiety, depression, and frustration. The ‘*F*’ category encompasses inquiries regarding daily functioning, including social activities, household tasks, and stress management. The questions in the ‘*C*’ category pertain to experiencing a serious illness or lack of control, etc. The total THI score can be calculated by adding up the points of the three subscales. Based on the total THI scores, five categories can be determined as follows: no handicap (0–16 points), ‘mild’ (18–36), ‘moderate’ (38–56), ‘severe’ (58–76), or ‘catastrophic’ (78–100) tinnitus handicap [22].

### 2.4. Statistical Analysis

All statistical methods were conducted using IBM SPSS version 25 software (IBM Corporation, Armonk, NY, USA). Simple linear correlation, Spearman’s correlation, and Pearson’s correlation tests were used to identify potential correlations between the parameters. Moreover, dot diagrams were created to illustrate the results. Furthermore, a linear regression model was used to analyse the influence of one parameter on another. The data did not follow a normal distribution, as determined by the Shapiro–Wilk test. Consequently, the Mann–Whitney *U* test, which is a non-parametric test, was used to identify any statistically significant differences. A *p*-value of less than 0.05 or 0.01 was considered statistically significant, respectively.

The audiometric edge frequencies were determined using various methods outlined in prior studies [9], with the most suitable method selected for each instance. The edge frequency was defined as the lowest frequency at which the hearing level (HL) reached or exceeded 20 dB, or approached 50 dB. This threshold indicates the point at which there is a transition from loss of outer hair cells to loss of inner hair cells [23]. Furthermore, the lowest frequency at which a difference in HL of more than 15 dB was observed was calculated [*HL* (*F2*) − *HL* (*F1*) > 15 dB] [24], according to Pan et al. When this difference could not be detected, the three lowest successive frequencies with a difference greater than 25 dB, [*HL* (*F3*) − *HL* (*F1*) > 25 dB], were calculated. If the frequency pairs could not be determined, the hearing loss edge was calculated at the lowest frequency where the difference in HL at adjacent frequencies was greater than at all other frequencies [*HL* (*F2*) − *HL* (*F1*) = max], as per Moore et al. [25].

## 3. Results

**Figure 1:**
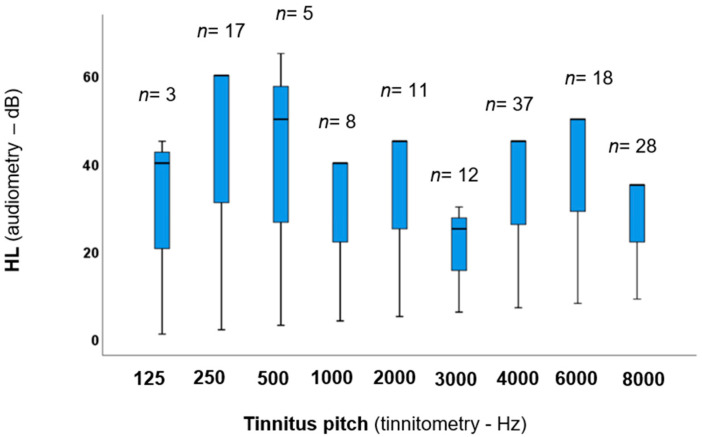
Boxplot displaying the tinnitus frequencies and HL. The numbers above the boxes indicate the number of participants in each group. The boxes represent the middle 50% of the data, and the whiskers represent the upper and lower 25%. The black line that divides the boxes into two parts indicates the median values. HL = hearing level.

Figure 1 presents the distribution of the tinnitus edge frequencies and the HL for each participant.

Based on Figure 1, the majority of participants were in the 4000 and 8000 Hz groups (27% and 20% of the total, respectively), suggesting that the tinnitus threshold was primarily detected at higher frequencies. The greatest HL was observed in the 250 Hz and 500 Hz frequency groups.

**Figure 2:**
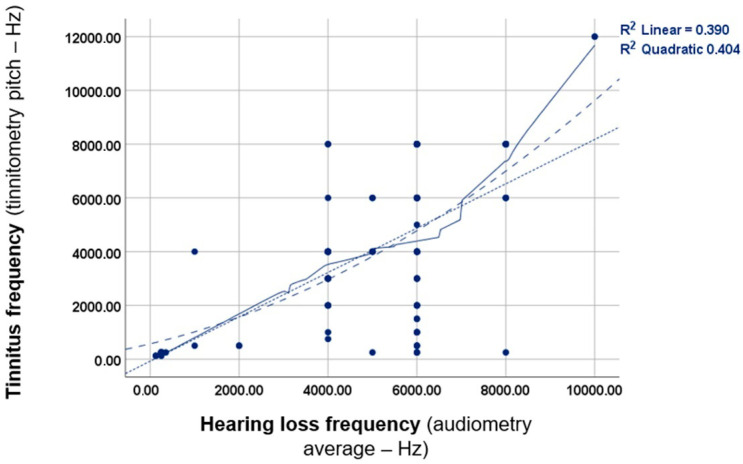
Simple linear correlation analysis to explore the relationships between tinnitus pitch and the average frequency of hearing loss, as determined by pure-tone audiometry Both linear and quadratic regression analyses were applied consistently throughout the research. The *R*^2^-value indicates the coefficient of determination for the linear correlation, and an *R*^2^-value close to 1 indicating a perfect correlation. The lines in the figure depict the fitted lines for linear and quadratic correlations.

Since one of the main purposes of the present work was to contrast the audiometry and tinnitus pitch matching results, their correspondence was initially analysed. The findings are summarised in Figure 2.

As Figure 2 reveals, a linear correlation was detected between tinnitus and hearing loss frequencies. This indicates that the pitch of tinnitus tends to increase with higher frequency hearing loss, and vice versa. Further statistical analysis using the Pearson’s correlation (*p* = 0.000 *; *rho* = 0.624) and Spearman’s tests (*p* = 0.000 *; *rho* = 0.549) revealed a statistically significant correlation between these two factors. These results emphasise that tinnitus and hearing loss affect the same frequency spectrum.

**Figure 3:**
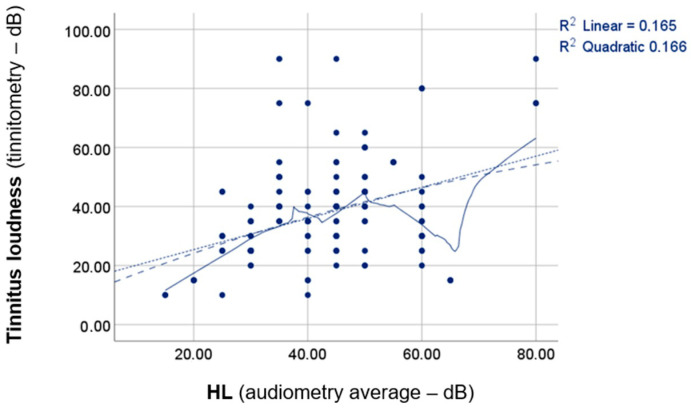
Simple linear correlation analysis between tinnitus loudness and HL. Linear and quadratic regression analyses were utilised. The *R*^2^-value indicates the coefficient of determination for the linear correlation, and an *R*^2^ of around 1 signifies a perfect correlation. The lines in the figure depict the fitted lines for linear and quadratic correlations. HL = hearing level.

The same analysis was conducted to examine the correlation between tinnitus loudness and HL. The results can be seen in Figure 3.

As illustrated by Figure 3, a slight (compared to Figure 1 and Figure 2) though noticeable correlation between tinnitus loudness and HL was observed. Both Pearson’s (*p* = 0.000 *; *rho* = 0.406) and Spearman’s (*p* = 0.000 *; *rho* = 0.375) tests confirmed this statistically significant relationship.

The influence of age and duration of symptoms was analysed in the next step. A linear regression model was applied, and the results are detailed in Table 1.

Table 1 presents data indicating that most parameters did not have a significant impact on the audiometry and tinnitometry results. However, age demonstrated a significant linear correlation with tinnitus loudness (*p* = 0.016 *) and HL (*p* = 0.000 *). This suggests that both tinnitus loudness and the severity of hearing loss tend to increase with age, as reflected by higher pure-tone thresholds. Therefore, it can be concluded that ageing affects the severity of tinnitus and HL. However, it does not significantly influence the pitch of tinnitus or the frequency of hearing loss. The loudness of tinnitus was significantly influenced by the duration of tinnitus symptoms (*p* = 0.022 *). Sex did not significantly affect tinnitometry or audiometry parameters.

Another linear regression model was developed to analyse the impact of tinnitus pitch, loudness, HL, and the frequency of hearing loss on THI scores. Additionally, the potential effects of age, sex, and duration of symptoms on THI outcomes were also explored. The results are given in Table 2.

In Table 2, it was noted that total THI scores were significantly affected only by the loudness of tinnitus (*p* = 0.021 *), while the pitch of tinnitus and audiometric parameters did not influence the scores. Hence, it can be concluded that the severity of self-reported tinnitus symptoms is determined solely by the loudness of tinnitus, rather than its frequency or hearing loss. Also, it demonstrates that sex significantly influenced total THI outcomes (*p* = 0.000 *), with higher THI scores noted in female patients (refer also to Figure 2 for the interpretation of the latter result). However, it is essential to recognise the unequal sex distribution in this study, as it may affect the results. Based on THI scores, age and symptom duration did not significantly affect the self-perceived severity of tinnitus.

**Figure 4:**
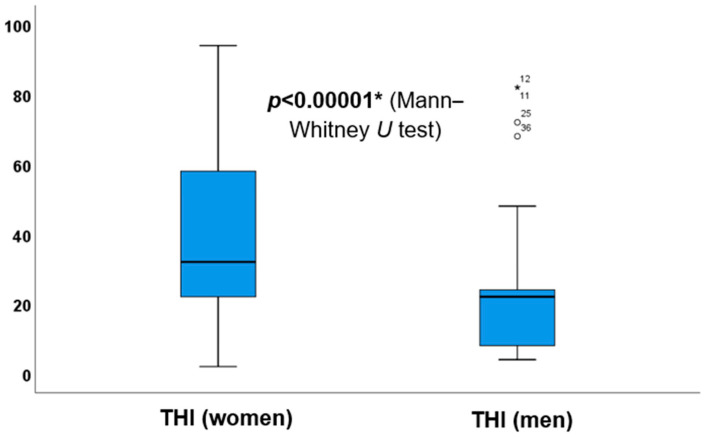
Comparison between total THI scores of male and female participants. The boxes represent the middle 50% of the data, and the whiskers represent the upper and lower 25%. The black line that divides the boxes into two parts indicates the median values. Differences were analysed using the Mann–Whitney *U* test (*p* < 0.05 *). THI = Tinnitus Handicap Inventory. The asterisk (*) denotes a statistically significant difference.

In Figure 4, the data indicate that female participants had higher total THI scores compared to their male counterparts. When comparing the total THI scores of male and female patients, a statistically significant difference was observed (*z*-score: 4.05, *p* < 0.0001 *), indicating that women had significantly higher scores.

## 4. Discussion

In the current study, numerous patients with chronic subjective primary tinnitus and associated hearing loss were included. A slight majority of the patients were female, and they exhibited significantly higher THI scores. The current study aimed to compare audiometry and tinnitometry results. The results showed a significant correlation between tinnitus pitch, hearing loss frequency, and tinnitus loudness and HL, respectively. This outcome suggests a potential link between their pathophysiology. The total THI scores were primarily influenced by the pitch of tinnitus, indicating that the frequency of the perceived sound significantly impacts the self-reported subjective tinnitus handicap. Among the general factors considered, such as the duration of symptoms, age, and sex, only age has had an impact on the duration of both tinnitus and HL. In contrast, the duration of symptoms influenced the loudness of tinnitus. This relationship may be attributed to changes in the auditory system over time, leading to chronic tinnitus. As a result, these factors do not impact the frequency of hearing loss or the pitch of tinnitus. The self-reported tinnitus handicap was primarily influenced by the loudness of tinnitus and the participants’ sex.

Previous studies have compared the profiles of audiometry and tinnitometry. For instance, one study found that only the frequency F50 (i.e., where HL was 50 dB) of hearing loss was correlated with the pitch of tinnitus. In that study, no correlation was observed between the edge frequency (i.e., the frequency at which hearing loss occurs) and the pitch of tinnitus. Interestingly, the pitch of tinnitus was recorded as being higher than the frequency of hearing loss, which contradicts the tonotopic model [6,9]. Therefore, the authors supported the homeostatic model. Their findings differ significantly from those of the current investigation, revealing a noteworthy correlation between edge frequencies and tinnitus pitch. Therefore, our study confirms the tonotopic model. A previous study concluded that the pitch of tinnitus fell within the frequency range of hearing loss, and no predictive frequency (i.e., F50) could be determined [26]. The findings of the current investigation generally align with prior research [23,25,26,27,28], highlighting a connection between tinnitus pitch and HL. However, some investigations did not find such a correlation [24,29,30]. Consequently, the literature presents conflicting data on this matter. Issues in calculating audiometry and tinnitometry parameters may contribute to these inconsistencies. Based on previous research methodologies, maximum or edge frequencies are typically determined. Various methods for calculating edge frequencies, derived from prior studies, are also available [23,24,25].

Another important issue to consider is the potential relationship between individuals’ perceptions of their tinnitus impact, as measured by the THI, and their audiometric test results. In the current study, it was found that THI scores were influenced solely by the loudness of tinnitus and the sex of the female participants. In agreement, a prior study found that patients with tinnitus and hearing loss had significantly higher TFI values compared to those without hearing loss. According to the authors’ recommendation, this should be considered for managing tinnitus. However, no correlation was found between the severity of tinnitus, age, and sex [14]. Another investigation found no significant correlation between subjective tinnitus distress (i.e., the THI scores) and audiological findings, regardless of whether tinnitus was accompanied by hearing loss. Consequently, the authors concluded that hearing loss could be a risk factor for tinnitus. Nevertheless, the distress caused by tinnitus is affected by various other factors, such as emotional aspects [15]. Another study found a significant correlation between THI and the visual analogue scale, as well as between tinnitus loudness and the visual analogue scale [30]. However, no correlation between THI and tinnitus loudness was defined, which is inconsistent with our investigation results. Additionally, the study did not observe any correlation with age and sex. A previous study also failed to find a correlation between psychoacoustic and subjective outcome measurements either; therefore, both should be used in everyday practice to quantify tinnitus [31]. A previous study has shown that there are more risk factors for tinnitus severity in women than in men. While age was not identified as an influencing factor, sex was found to play a significant role. The study explained these sex differences through various influencing factors that affect men and women differently. The severity of tinnitus in women is influenced by several factors, including the type of tinnitus sound, sensation level, results from the residual inhibition test, locations of tinnitus, and the degree of hearing loss. In contrast, for men, only the types of tinnitus and the degree of hearing loss were found to affect the severity of tinnitus [32]. Sex differences in tinnitus may result from morphological and functional changes in the auditory system, influenced by hormonal interactions [33]. Furthermore, brain activity associated with tinnitus differs by sex, with women exhibiting more pronounced changes in cortical activity [34]. Another investigation found no significant difference in THI scores between men and women; however, women reported higher levels of depression, anxiety, and worries [35]. As a result, there are inconsistent findings regarding tinnitus severity in relation to sex differences. The potential influence of hormones on tinnitus has yet to be confirmed. Research on animals indicates that administering oestrogen and progesterone may cause physical and functional damage in the cochlea [36]. Therefore, sex differences in tinnitus may be linked to hormonal fluctuations.

The findings from this study support the tonotopic model of tinnitus, indicating a plastic reorganisation in central pathways after hearing loss [6]. This model outlines the connection between the frequency of tinnitus sounds and the pitch perceived by the individual. A previous study highlighted the impact of tinnitus on plastic reorganisation in the central nervous system. The research found that individuals with hearing loss and tinnitus exhibited less reorganisation in the auditory cortex compared to those with hearing loss but no tinnitus. As a result, the authors suggest that tinnitus is not associated with significant plastic reorganisation; rather, it stems from an impaired adaptation mechanism [37]. An investigation has shown that neural reorganisation contributes to the development of tinnitus. Through auditory brainstem response audiometry, researchers found a significant difference in the amplitude ratio of the III/I, V/I, and V/III waves between tinnitus and non-tinnitus patients. Specifically, patients with tinnitus exhibited higher amplitude ratios in these waves. Furthermore, it was observed that tinnitus distress (i.e., THI scores) is related to the functioning of the central auditory system [38].

The present study had some limitations. First, there is a lack of using objective audiometric testing. Additionally, a potential limitation of pure-tone audiometry is that frequencies above 8000 Hz were not assessed, even though the frequency spectrum used reflects the methods commonly employed in everyday practice. Moreover, a consensus has yet to be reached on the calculation of the edge frequency. Therefore, different methods, such as those in previous investigations, were used in our study. Additionally, the unequal distribution of sexes may introduce bias in the findings related to sex differences.

## 5. Conclusions

The current investigation suggests a correlation between tinnitus and hearing loss parameters, indicating a tonotopic model behind tinnitus complaints Additionally, the correlation between tinnitus loudness and severity, as measured by THI scores, emphasises the link between tinnitus intensity and self-reported handicap. Therefore, it is highly recommended to use the THI questionnaires in everyday practice. However, when assessing individual patient complaints in clinical practice, other parameters such as tinnitus pitch and hearing loss should also be considered.

## Figures and Tables

**Table 1:** A linear regression model to analyse the influence of age, duration of symptoms, and sex on tinnitus and HL characteristics. A *p*-value of less than 0.05 * was considered statistically significant. Std. = standard; HL = hearing level. The asterisk (*) denotes a statistically significant difference.

Model	Dependent Variable	Unstandardised Coefficients		Standardised Coefficient	*t*	*p*-Value
		*β*	Std. Error	*β*		
Age	Tinnitus pitch	1.063	18.593	0.005	0.057	0.954
	Tinnitus loudness	0.259	0.107	0.213	2.434	0.016 *
	HL	0.509	0.073	0.543	6.942	0.000 *
	Hearing loss frequency	15.002	14.075	0.098	1.066	0.288
Duration	Tinnitus pitch	8.884	5.726	0.143	1.552	0.123
	Tinnitus loudness	0.076	0.033	0.203	2.317	0.022 *
	HL	0.001	0.023	0.005	0.652	0.951
	Hearing loss frequency	3.600	4.334	0.077	0.831	0.408
Sex (0 = women, 1 = men)	Tinnitus pitch	−149.403	502.156	−0.026	−0.298	0.767
	Tinnitus loudness	0.205	3.036	0.006	0.067	0.946
	HL	511.987	377.722	0.116	1.355	0.178
	Hearing loss frequency	3.235	2.319	0.119	1.395	0.165

**Table 2:** A linear regression model to analyse the impact of age, duration of symptoms, and sex on total THI scores. A *p*-value less than 0.05 * was considered statistically significant. Std. = standard; THI = Tinnitus Handicap Inventory. The asterisk (*) denotes a statistically significant difference.

Model	Dependent Variable	Unstandardised Coefficients		Standardised Coefficient	*t*	*p*-Value
		*β*	Std. Error	*β*		
Tinnitus pitch	THI	0.001	0.001	0.121	1.111	0.269
Tinnitus loudness		0.293	0.125	0.217	2.344	0.021 *
Hearing loss frequency		0.001	0.001	0.070	0.642	0.522
HL		−0.130	0.162	−0.074	−0.799	0.426
Age		−0.039	0.153	−0.024	−0.254	0.800
Sex (0 = women, 1 = men)		−15.208	3.863	−0.320	−3.937	0.000 *
Tinnitus duration		0.05	0.047	0.010	0.113	0.910

## Data Availability

The data presented in this study are available on request from the corresponding author.
